# Climate Change and Macro-Economic Cycles in Pre-Industrial Europe

**DOI:** 10.1371/journal.pone.0088155

**Published:** 2014-02-07

**Authors:** Qing Pei, David D. Zhang, Harry F. Lee, Guodong Li

**Affiliations:** 1 Department of Geography, The University of Hong Kong, Hong Kong; 2 International Centre for China Development Study, The University of Hong Kong, Hong Kong; 3 Department of Statistics and Actuarial Science, The University of Hong Kong, Hong Kong; University of Oxford, United Kingdom

## Abstract

Climate change has been proven to be the ultimate cause of social crisis in pre-industrial Europe at a large scale. However, detailed analyses on climate change and macro-economic cycles in the pre-industrial era remain lacking, especially within different temporal scales. Therefore, fine-grained, paleo-climate, and economic data were employed with statistical methods to quantitatively assess the relations between climate change and agrarian economy in Europe during AD 1500 to 1800. In the study, the Butterworth filter was adopted to filter the data series into a long-term trend (low-frequency) and short-term fluctuations (high-frequency). Granger Causality Analysis was conducted to scrutinize the associations between climate change and macro-economic cycle at different frequency bands. Based on quantitative results, climate change can only show significant effects on the macro-economic cycle within the long-term. In terms of the short-term effects, society can relieve the influences from climate variations by social adaptation methods and self-adjustment mechanism. On a large spatial scale, temperature holds higher importance for the European agrarian economy than precipitation. By examining the supply-demand mechanism in the grain market, population during the study period acted as the producer in the long term, whereas as the consumer in the short term. These findings merely reflect the general interactions between climate change and macro-economic cycles at the large spatial region with a long-term study period. The findings neither illustrate individual incidents that can temporarily distort the agrarian economy nor explain some specific cases. In the study, the scale thinking in the analysis is raised as an essential methodological issue for the first time to interpret the associations between climatic impact and macro-economy in the past agrarian society within different temporal scales.

## Introduction

Climate change has been proven to be the ultimate cause of social crisis in pre-industrial Europe at a large scale [1]. However, detailed analyses on climate change and macro-economy cycle in the pre-industrial epoch remain lacking, especially within different temporal scales. Generally, numerous theories of economic cycle can be divided into two main categories, namely, supply-led (e.g., monetarism and real business cycle theory) and demand-led (e.g., Keynesian economics and new classical economics) [2]. Moreover, certain social factors have also been adopted to explain economic cycles, including population [3] and war [4]. However, cycle theory has not been widely accepted in economics because the statistical materials and satisfactory explanations on fluctuations (price and inflation) are limited [5].

Although agrarian economy was discussed as an important transitional mechanism linking climate change and social crisis by Zhang et al. [1], certain issues pertinent to the relationships of macro-economic cycles under climate change at a large scale remain unresolved. First, the systemic associations between climate change and macro-economy cycle in the pre-industrial epoch have not been analyzed in detail. Second, Zhang et al. only quantitatively studied the low-frequency linkages (low-pass filtered data) in the conceptual model. The high-frequency patterns remain unknown. Third, how precipitation affects the agrarian economy at a large scale is not discussed, particularly in a quantitative manner.

In the present study, these remaining issues were quantitatively analyzed. The association between climate change and cycle of agrarian economy was scrutinized with the original data, low-frequency filtered data, and high-frequency filtered data in this study. Compared with the study of Zhang et al., additional economic indicators (real wage and CPI) were added as the most important indicators to reflect social well-being [6] together with a new climatic indicator, precipitation. Then, systematical connections between climate change and macro-economic cycles in pre-industrial Europe were built. Furthermore, the dual role of population size (producer/consumer) in the agrarian economy was discussed within different temporal scales.

As the basis of agrarian economy in the pre-industrial era, agricultural production was extremely dependent on climate [7]. Logically, climate change affects the agrarian economy via the supply side. During the last half of the century, climatic fluctuations have been attached with great importance to economic growth [8]. However, the associations between climate change and the agrarian economy were even closer in pre-industrial society. Numerous discussions on the climatic effect on the grain production in agrarian economy have been made, but they are somehow associated with various limitations. For instance, certain studies have only focused on extreme events and consequently, produced biased findings [9]. Others have confined the study scope to a particular country, such as England [10], Poland [11], Germany [12], France, and Russia [13]. However, complex interactions between nature and society should be disentangled at a large spatial scale [14], [15]. Particularly, the continental integration of the grain market in Europe started in the early modern period [16], [17]. Furthermore, the research span of existing studies is also relatively short [18], [19]. A study period that will not extend to the centenary level will hardly reflect the climatic effect, because the minimum time to observe climate change is 30 years [20].

Subject to these deficiencies, the present study aims to uncover the systematic structure between climate change and macro-economy cycle in agrarian Europe, which has the most detailed records on climate [21] and crop cultivation [22]. The study scale is set as the whole of Europe (including all countries) and delimited our study period from AD 1500 to AD 1800, with the advantage of having relatively rich data. Our chosen timespan is nested within the Little Ice Age with the lowest temperature throughout the past millennium [23]. This period also encompassed the decline of the macro-economy and the General Crisis of the 17^th^ Century [24]. Since the 18^th^ century, when temperature was increasing, the output of the agrarian economy recovered and increased [25] to form at least one complete macro-economic cycle within the study period [26].

A typical time series of macro-economic cycle is composed of the elements of low-frequency trends (long run) and high-frequency fluctuations (short run) [27], which contain unique information at diverse temporal scales [28] the same as climate. Climate fluctuation that endures 30 years or more is usually defined as climate change with a long-run trend (low-frequency) [20]. The rest of short-run fluctuations (high-frequency) is only considered climate variations [29]. Regardless of how the data curve appears in the temporal domain, both high- and low-frequency information can be found if a certain mathematical method from the frequency domain is applied in the analysis. In the study, the Butterworth filter was adopted to smooth the data series into long-term trend (low-frequency) and short-term fluctuations (high-frequency). Granger Causality Analysis (GCA) was applied to scrutinize the association between climate change and macro-economy at different frequency bands. Lag calculation in GCA can consider the lasting dynamics of climatic effect and auto-correlation [8], [29]. GCA was also applied by Zhang et al. [1], and it is a widely accepted method of building a causal relationship [30]. Therefore, GCA was adopted in the present study to investigate the associations between climate change and macro-economic cycle in the pre-industrial Europe.

The findings were all identified according to the quantitative results in the study. We claimed that climate change can only show its significant effect on macro-economic cycle in the pre-industrial Europe in the long run, because the term “climate change” only reflects macro-climatic trend. However, human society can relieve the short-term influence of climate variations by social adaptation methods and self-adjustment mechanism. Temporal-scale thinking is fundamentally important to interpret climatic effect and macro-economy in the past agrarian society. Our goal in this study was implemented with three hundred years and on a continental scale to reason a general rule and trend on climate change and macro-economy. Therefore, attention was focused neither to individual incidents that can temporarily distort the agrarian economy nor to explain some certain specific cases. Despite limitations, we believe that this broad-brush approach suits the scope of the present study to quantitatively generate an association between climate change and the agrarian economy in the past society at a large spatial and long-term scale.

## Materials and Methodology

**Figure 1:**
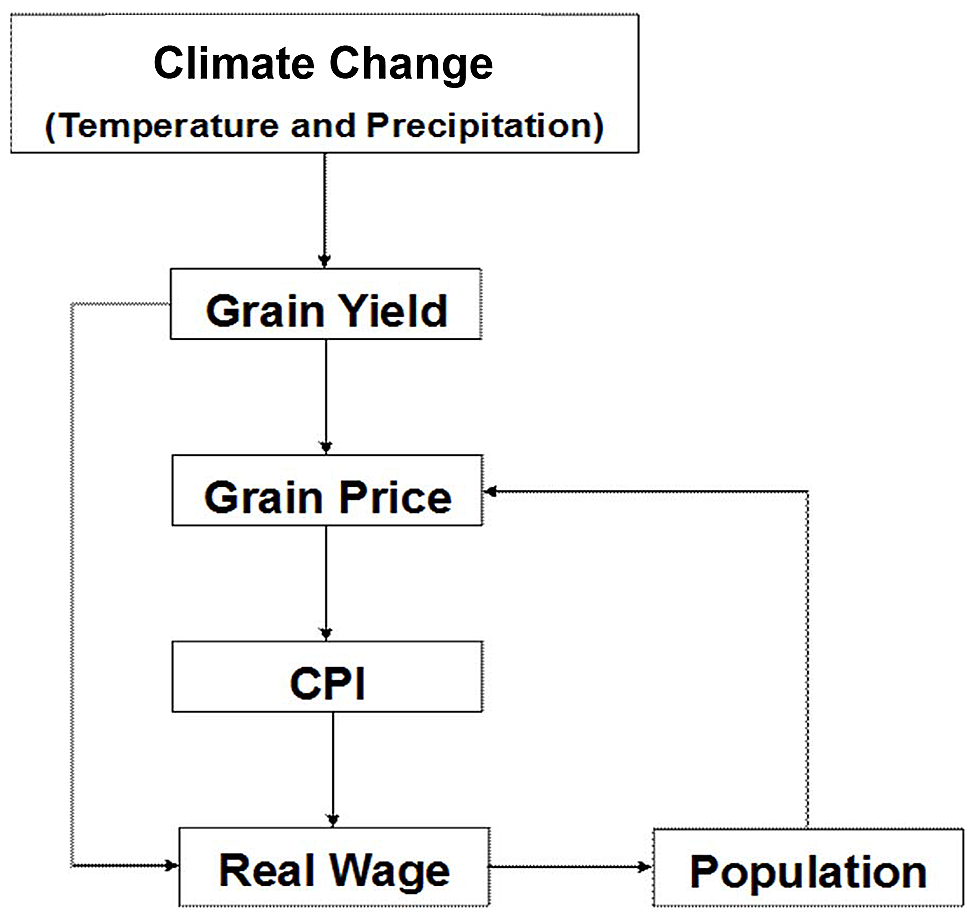
Conceptual Model of Climate Change and Macro-Economy Cycles in Pre-Industrial Europe. Note: The arrows should be read as: “Change in *X* is associated with change in *Y*”.

The associations between climate change and macro-economic system were constructed as shown in Figure 1. This conceptual framework was based on the conceptual model by Zhang et al. [1], as well as the commonly accepted theories from social sciences and economics, which will be specifically illustrated in the following sections. The conceptual model (Figure 1) in this study has referred to Zhang et al. and other theories, but the study constructed a more insightful relationship between climate change and macro-economic cycle by adding more indicators.

### 2.1 Temperature and Precipitation

Precipitation was excluded in previous studies about climate change and social crisis [1], [31]. Precipitation was taken into the analysis to advance the findings regarding the relations between climate change and society. In the research, anomaly series of temperature and precipitation in Europe were derived from the latest reconstructions based on tree-rings [32], of which the data have the high resolution at the annual scale. The reconstruction has high reliability because of its large sample basis. A total of 1,546 tree-ring width series were used for annual temperature reconstruction and 7284 series for precipitation reconstruction yearly [32].

### 2.2 Grain Yield (Bio-Productivity)

Yield ratio is a useful assessment of economic productivity [33]. In an agrarian society, yield ratio reflects the capacity of social production and is important in the economy [34]. Our grain yield data were derived from the dataset of van Bath [35], which is calculated as follows:
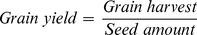
(1)


The yield refers to the arithmetically averaged yield of four “small grain” crops, namely, wheat, rye, barley, and oats. The dataset of van Bath [35] contains the yield ratio of 18 countries in pre-industrial Europe. The data were obtained by arithmetically averaging the grain yield ratios of these countries.

### 2.3 Grain Price

Our grain price series was derived from the website of the International Institute of Social History (http://www.iisg.nl/hpw/data.php#europe). The price data covered four types of grains (wheat, rye, barley, and oats) in 16 major European regions. The unit is “grams of silver per liter.” Grain is a basic necessity for human consumption, for which no good substitute exists, especially during the pre-industrial period in Europe [36]. The continental integration of grain market in Europe started in the study period [16], [17]. It has the theoretical basis to aggregate a combined price of grain in Europe. The prices of wheat, rye, barley, and oats were calculated to construct the grain price series. Any missing data were linearly interpolated to give an annual time series. Then, the annual price series of the four types of grains was arithmetically averaged for use in this study, which can eliminate noise and achieve a more accurate trend.

However, little can be done to dampen price volatility given the high costs of inter-temporal and spatial transport [37], and the agrarian economy was barely affected by intercontinental trade. Government intervention in the past can relieve recurrent price shocks caused by temporary grain shortages [38], but it is of minimal help if the grain shortage is at a global or large regional scale [39].

### 2.4 Consumer Price Index

The consumer price index (CPI) measures the cost for a typical family to buy a representative basket of goods for daily needs, including wheat, barley, oats, rye, beef, peas, cheese, eggs, oil, honey, coal, beans, beef, sugar, and butter. CPI is also referred to as the cost-of-living index [6]. CPI data were obtained from the International Institute of Social History and Allen-Unger Global Commodity Prices Database (http://www.history.ubc.ca/faculty/unger/ECPdb/index.html).

### 2.5 Real Wage

Real wage refers to income after considering the effects of inflation on the purchasing power of nominal wage. Real wage is viewed as an indicator of past welfare conditions [40], [41]. Our real wage index was derived from two datasets. The first dataset consists of the real day wages of farm laborers in England [41]. The second dataset was compiled by Allen and consists of the real wages of building craftsmen and laborers in 19 major European cities [42]. The real wages of farm laborers are in decadal units, and those of the building artisans and laborers are in annual units. Therefore, the latter was transformed into identical decadal units. Each real wage series was normalized to homogenize the original variability of all series. Finally, the two normalized series were arithmetically averaged and linearly interpolated to create an annual wage index series.

Nominal wage is positively determined by social productivity [41], which is measured by grain yield in the agrarian society. Therefore, in the pre-industrial era, real wage is co-decided by grain yield positively and inflation level (CPI) negatively. With the decline in real wage, the population can suffer from undernourishment and even famine because people cannot afford food. Endemic diseases among the population may increase virulence and contagion [43]. Subject to the reduction of real wage, people tend to postpone marriage or have fewer children and the net outcome is an excess of deaths over births and a reduction in aggregate population [44], [45]. Therefore, real wage is positively correlated with population growth.

### 2.6 Population Size

This study does not represent formal demographic research. However, we prefer to include population as an independent variable in the economy system to follow the framework of previous Zhang et al’s study [1]. The increase and decline in population largely influence the economy as well. In the current study, the population of Europe was extracted from the *Atlas of World Population History* by McEvedy and Jones [46]. The effects of migration on population changes at this continental scale were neutralized, because the whole Europe is set as the study area. Moreover, the common logarithm of the data points was taken, linearly interpolated, and anti-logged back to create an annual time series, because the population data were taken at irregular time intervals. This method prevents any distortion of the population growth rate as a result of the data interpolation. Despite the coarse feature of population data series, both high- and low-frequency information can be found by applying the Butterworth filter, which is a mathematical method from the frequency domain (c.f. Section 1).

Time-series with obvious long-term trends, such as grain price, CPI, real wage, and population size, was linearly detrended previously to elicit the “real association” among the assorted variables [3], [7].

## Methods

### 3.1 Smoothing-Butterworth Filter

Both climate change and economic cycle consist of slowly evolving trend and rapidly varying components (c.f. Section 1). To obtain the trend estimation in the time series, a low-pass filter was adopted as a technique in the analysis [47]. Among different kinds of filters, the Butterworth filter is commonly used to smooth the data series. Particularly, the Butterworth filter can also perform better with business-cycle estimation to decrease the risk of inducing spurious results [48]. Furthermore, the Butterworth filter can flexibly allow the smoothed cycles to be extracted from the economic time series [49].

Based on the research about climate change in Europe from AD 1766 to 2000, European seasonal temperature and precipitation indicate a trend of approximately 40 years [50]. In a previous study, a 40-year low pass filter was used to filter the data to check the general trend of climate change [51]. Accordingly, the Butterworth filter was set to 40 years for low-pass and high-pass filter.

**Figure 2:**
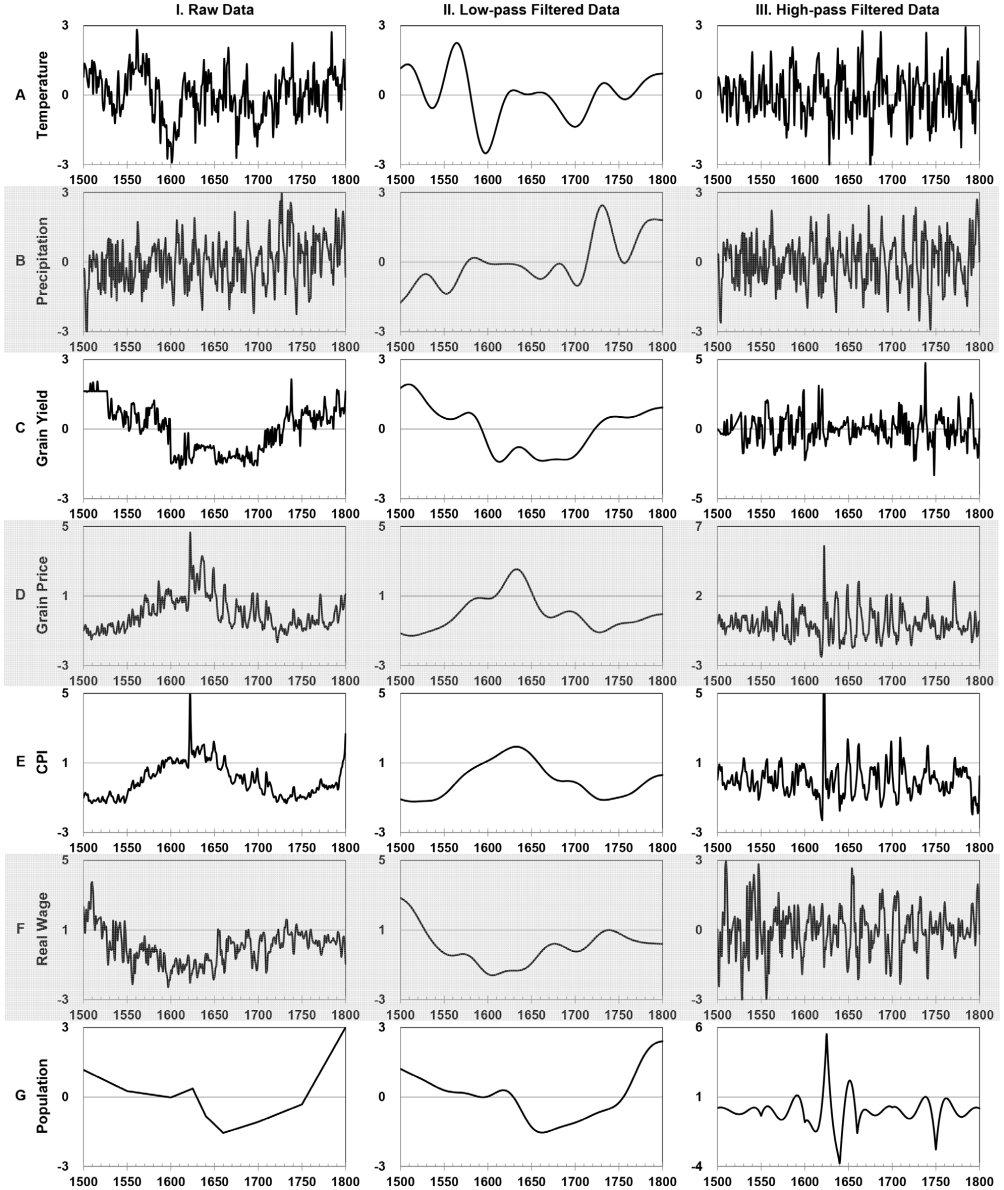
Visualization of the Causal Linkages in the Conceptual Model of Raw, Low-Pass Filtered, and High-Pass Filtered Data. Note: Column I represents raw data; Column II represents low-pass filtered data; and Column III represents high-pass filtered data. Row (a) represents Temperature; (b) Precipitation; (c) Grain Yield; (d) Grain Price; (e) CPI; (f) Real Wage; and (g) Population. Variables with obvious long-term trends, such as grain price, CPI, real wage, and population size, were linearly detrended. All data have been standardized.

The low-pass filtered data of climate represent the climate change in a macro-trend, and the low-pass filtered economic data series characterizes the long-term economic trend. The high-pass filtered data of climate signify the climate variations, and the high-pass filtered economic data depict the random high-frequency fluctuations in the macro-economy. The original climatic and economic data include a mixed signal of long-term trend and short-term fluctuations. Figure 2 shows the raw data, low-pass filtered data, and high-pass filtered data based on 40 years.

### 3.2 Verification of the Causal Linkages

GCA is an effective method in building a causal relationship [30]. GCA does not definitely suggest causality, but it is a method that quantitatively supports theoretical analysis with statistical evidence [1]. In addition, statistical laws are considered important in interpreting historical laws based on numerous cases, even though statistical laws cannot be applied to every case [52]. GCA was carried out between variables with zero mean [53], and thus the variables for GCA in the study were standardized first. Prior to GCA, the Augmented Dickey-Fuller (ADF) test was necessarily employed to check the stationarity of the time-series as the precondition assessment. If necessary, differencing was used to transform the time-series into stationary ones [54]. The lag was set in two ways: the first was based on the theoretical and empirical knowledge of the relationships, such as instantaneous cause; the second was based on statistical criteria for others [55], [56]. Akaike’s information criterion (AIC) was adopted to determine the appropriate lag length [57] as the statistical criteria. The detailed calculation process of GCA is shown in File S1. Based on lag selections following theoretical background or statistical criteria, we categorized our causal linkages (null hypotheses) into two groups as follows:

[Group 1]

1. Temperature does not Granger-cause Grain yield
2. Precipitation does not Granger-cause Grain yield
3. Grain yield does not Granger-cause Grain price
4. Population size does not Granger-cause Grain price.

[Group 2]

1. Grain price does not Granger-cause CPI
2. Grain yield does not Granger-cause Real wage
3. CPI does not Granger-cause Real wage
4. Real wage does not Granger-cause Population size.

For causal linkages in Group 1, the relationship materializes almost instantaneously and the time lag for GCA was set to 1 [1]. For Group 2, the associations between two variables indicates time lag. Therefore, AIC statistical criterion was calculated as the time lag for GCA on the linkages in Group 2. The ADF Test and AIC lag information are shown in Table S1–S9 in File S1. After setting the lag length, each pair of time-series with a causal relationship was checked at the same level of differencing under the precondition of stationarity. Table 1 shows the correlation results of each link by raw data, low-pass filtered data, and high-pass filtered data. Only the results of raw and low-pass filtered data followed the conceptual model. Table 2, Table 3, and Table 4 separately list the results of GCA on raw data, low-pass filtered data, and high-pass filtered data, respectively.

**Table 1:** Correlation Analysis Results of Causal Linkages in Figure 1.

	Raw Data	Low-passData	High-passData
**[Group 1]**			
(1) Temperature– Grain yield	0.356**	0.533**	0.060
(2) Precipitation – Grain yield	0.032	0.092	0.020
(3) Grain yield – Grain price	−0.486**	−0.621**	−0.118*
(4) Population size– Grain price	−0.127*	−0.203**	0.118*
**[Group 2]**			
(5) Grain price– CPI	0.917**	0.966**	0.807**
(6) Grain yield – Real wage	0.524**	0.697**	−0.001
(7) CPI– Real wage	−0.745**	−0.841**	−0.588**
(8) Real wage– Population size	0.222**	0.273**	−0.049

*Notes*: Significance (2-tailed):

*p<0.05,

**p<0.01.

**Table 2:** GCA Results for Each of the Causal Linkages by Raw Data.

Null hypothesis about causal linkages	F	p
**[Group 1]**		
(1) Temperature does not *Granger-cause* Grain yield△	6.047	0.015*
(2) Precipitation does not *Granger-cause* Grain yield△	0.134	0.714
(3) Grain yield does not *Granger-cause* Grain price△	0.943	0.332
(4) Population size does not *Granger*-*cause* Grain price#	2.866	0.092∧
**[Group 2]**		
(5) Grain price does not *Granger-cause* CPI△	4.105	0.017*
(6) Grain yield does not *Granger-cause* Real wage△	0.789	0.627
(7) CPI does not *Granger-cause* Real wage△	1.985	0.041*
(8) Real wage does not *Granger-cause* Population size†	Nil	

*Notes*:

†For 0 AIC lag of population, we exclude link (8) from GCA due to data limitation. Differencing:

△ no difference,

# 2nd difference. Significance (2-tailed):

∧ p<0.1,

*p<0.05,

**p<0.01,

***p<0.001.

**Table 3:** GCA Results for Each of the Causal Linkages by Low-Pass Filtered Data.

Null hypothesis about causal linkages	F	p
**[Group 1]**		
(1) Temperature does not *Granger-cause* Grain yield△	120.902	0.000***
(2) Precipitation does not *Granger-cause* Grain yield△	18.579	0.000***
(3) Grain yield does not *Granger-cause* Grain price△	22.464	0.000***
(4) Population size does not *Granger*-*cause* Grain price△	67.664	0.000***
**[Group 2]**		
(5) Grain price does not *Granger-cause* CPI△	7.376	0.000***
(6) Grain yield does not *Granger-cause* Real wageα	1.994	0.016*
(7) CPI does not *Granger-cause* Real wage△	1.855	0.028*
(8) Real wage does not *Granger-cause* Population sizeα	1.818	0.033*

*Notes*: All data series were filtered by 40-yr Butterworth low-pass filter prior to statistical analysis. Differencing:

△ no difference,

α 1^st^difference. Significance (2-tailed):

∧ p<0.1,

*p<0.05,

**p<0.01,

***p<0.001.

**Table 4:** GCA Results for Each of the Causal Linkages by High-Pass Filtered Data.

Null hypothesis about causal linkages	F	p
**[Group 1]**		
(1) Temperature does not *Granger-cause* Grain yield△	2.661	0.104
(2) Precipitation does not *Granger-cause* Grain yield△	0.064	0.800
(3) Grain yield does not *Granger-cause* Grain price△	1.382	0.241
(4) Population size does not *Granger*-*cause* Grain price△	0.804	0.371
**[Group 2]**		
(5) Grain price does not *Granger-cause* CPI△	0.824	0.482
(6) Grain yield does not *Granger-cause* Real wage△	1.512	0.135
(7) CPI does not *Granger-cause* Real wage△	1.177	0.307
(8) Real wage does not *Granger-cause* Population size△	0.527	0.871

*Notes*: All data series were filtered by 40-yr Butterworth high-pass filter prior to statistical analysis. Differencing:

△ no difference,

α 1^st^difference. Significance (2-tailed):

∧ p<0.1,

*p<0.05,

**p<0.01,

***p<0.001.

## Results and Discussion

### 4.1 Associations between Climate Change and Macro-Economic Cycle at Different Temporal Scales

Only low-pass filtered data (low frequency) were adopted in the study of Zhang et al. Without a full view of the raw and high-frequency data, how macro-economic cycle reacts to climate change cannot be addressed clearly in different temporal scales. Therefore, the study improves the understanding on climate change and social responses within different temporal scales (frequency bands) from the perspective of agrarian economic cycle under climatic effect.

As addressed in Section 1, the low-pass filtered data represent the long-term trend of data series, whereas high-pass filtered data are the short-term fluctuations. Raw data include all mixed information about both the evolving trend and varying elements. Based on the statistical results, all links in the conceptual model pass GCA with low-pass filtered data in Table 3. Only several links of the raw data pass GCA in Table 2, whereas no links of high-pass filtered data pass GCA as shown in Table 4. In this sense, the conceptual model, which was constructed based on theories, only works in the long term, whereas none of the links works in the short term. Based on the GCA and correlation analysis results, we obtain the below implications within different temporal scales.

First, based on GCA results in Table 3, the effect of climate change (in the long run) can only be observable to macro-economic cycle by affecting supply side, that is, agricultural output. As a determinant factor in agricultural production in the pre-industrial Europe, climate change should not be neglected from macro-economy; human society will experience an unfortunate period if the deteriorated climate change lasts for a long time. The worst event is harvest failure, which could last for years. Although humans could relieve the climatic effect in the long run by adaptation, institutional and social buffering mechanisms will be ultimately exhausted by the recurrent subsistence crises caused by long-term cooling [58]. Worldwide empirical studies have also revealed that social buffers ultimately become ineffective and unable to prevent social crisis during persistent agricultural shortages induced by long-term cooling [1], [39]. Associations between climate change and macro-economy in pre-industrial era are convincing only in the long term.

Second, based on the GCA results by raw data in Table 2, the high-frequency fluctuations can be prevented from causing decisive disturbances on the linkages only if the trends of linked variables were strongly related. The significant links in Table 2 have strong associations that can almost be treated as laws. Aside from these links in Table 2, the other links were widely accepted in theory, but the GCA results are not significant because of the strong disturbance of high-frequency fluctuations. Hence, the study scale should be seriously perceived, even for the commonly accepted rules.

Third, although agricultural production was highly dependent on climate in the past, society can relieve the effect of climate variations (at a short term) as the GCA results of high-pass filtered data in Table 4. The p-value of GCA result of temperature-yield is 0.104 and precipitation–yield is 0.800, which justifies the social capacity to mitigate the influence of climate variations. Facing short-term climate variations, the adaptation methods, such as labor and land input, can maintain the yield level [58], [59]. Furthermore, the grain storage can also partly stabilize the grain price in the market in spite of low yield ratio [60]. Climate variations are not the direct trigger of economic cycle in short term. Besides, the economic mechanism can also be kept stable despite the sudden shocks among each inner element based on quantitative results of GCA in Table 4. All these links are widely accepted in economic theories (c.f. Section 2). Therefore, the short-term social capacity is effective in relieving the influence of climate variations based on GCA results.

### 4.2 Control Factor of Climate Change in Pre-Industrial Europe - Temperature

Precipitation was excluded in previous studies on climate change and social crisis [1], [31]. Typically, temperature and precipitation are the two chief climate variables included [61]. By including precipitation in the study, the climatic effect (cooling and drying) on the agrarian economy were interpreted more deeply.

Based on Section 4.1, the climatic effect on the macro-economic cycle can possibly be examined at a long-term scale. According to Table 3, both temperature and precipitation are significant in the GCA results. However, temperature has a higher significance than precipitation according to a statistical *F*-test. The importance of temperature can be further supported by GCA on raw data in Table 2. In the meantime, the result of correlation analysis between low-pass filtered temperature and glacier fluctuations [62] is 0.499, which is significant at the level of 0.01 and justifies the low-frequency changes in temperature (Fig S1 in File S1). The connection of trends between temperature and grain yield (economic productivity) is too strong to be disturbed by the high-frequency fluctuations. The p-value of GCA on temperature and grain yield is 0.104, which is at the threshold of 0.1 significant level, even in Table 4 that shows high-pass filtered data. Moreover, only the correlation between precipitation and yield is insignificant for three pairs in Table 1.

Our research was implemented at the continental scale. This spatial scale can lead to the finding that temperature is the controlled climatic factor of macro-economic cycle in pre-industrial Europe. The results in the study are also consistent with past findings, that is, temperature, instead of precipitation, more closely affects the economy [16], [29]. Temperature is a more appropriate indicator of climate change at a large spatial scale [63]. The sufficient moisture in the air guarantees abundant precipitation throughout Europe, but results in less effective warming because of latitudinal location [64]. However, the importance of precipitation in agriculture at the regional or local scale cannot be denied.

### 4.3 Supply-Demand Mechanism in Pre-Industrial Macro-Economy

In the analysis, an interesting result with regard to population and price is shown in Table 1. Population and price are negatively correlated based on raw and low-pass filtered data in the long trend. The high-pass data of population and price are positively correlated after excluding the long trend in the time series, which implies the changed role of population at different temporal scales.

Population has two roles, namely, producer and consumer, in the agrarian economy. The dual role of population (producer/consumer) has never been discussed in any previous study about the pre-industrial Europe, especially within different temporal scales. Similarly, Zhang et al. ignored the dual role of population. This blindness is also partly caused by the ignorance of temporal-scale thinking.

In pre-industrial society, farming requires intensive labor input, which accounts for 80 percent of the population [65], [66]. As a producer, population is negatively correlated with price, because the larger population size can produce more supply to decrease price. However, as consumers, population can add burden on the demand side to increase price. Therefore, the correlation results of raw and low-pass filtered data indicate that the population is mainly the producer. By contrast, the population acts as a short-term consumer, according to the correlation result of high-pass filtered data. Overall, the role of the population as the producer is more significant than its role as the consumer because of the negative correlation results of raw data during the study period.

## Conclusions

This study finds that climate change is more substantial in leading the cycles of macro-economy in the long term. In the short term, human society can relieve the influence of climate variations on agriculture production and agrarian economy by adaptation methods, such as self-adjustment mechanism. In the short term, the economy in pre-industrial Europe can maintain stability despite the climatic effect. Moreover, temperature, rather than precipitation, is the controlled factor in the pre-industrial macro-economy at the large spatial scale. By examining the supply-demand mechanism in the grain market, population acted as the consumer during the study period and in the short term, whereas it acted as the producer in the long term. In sum, temporal-scale thinking deserves attention in interpreting the associations between climate change and macro-economy in the past agrarian society according to all the quantitative analysis results in the study. Temporal-scale thinking was raised as an essential methodological issue for the first time in the present study to interpret the associations between climatic impact and macro-economy in the past agrarian society.

Besides, GCA is only adopted to testify the “statistical law” in our study. Still, statistical laws are considered important in interpreting historical laws based on numerous cases but may not apply to every single case [52]. Statistical methods are adopted as the official mathematical language to construct causal relationships by inferring major connections [67]. According to our study, a causal or statistical causal link can only be constructed in the long term and at a large spatial scale. The causality analysis should be combined with scale thinking as well. However, we do not deny the significance of other causality analysis, such as Mackie’s INUS causality [68], [69], which might be more reliable for small-scale studies or those investigating specific cases.

Past climate changes are etched on the landscape and a subtext of our economic and social history [9]. Aside from adopting an economic perspective to examine the macro-economy, the effect of climatic force should be also considered when interpreting the fluctuations of the agrarian economy in European history. However, scholars should keep climate change in mind as they consider “human history” given that climatic anomalies and declines in temperatures often have had catastrophic consequences for the macro-economy [70].

## Supporting Information

File S1 **This file contains Tables S1–S9 and Figure S1.** Table S1, ADF Test for the Variables of Raw Data in Group 1 (Lag = 1). Table S2, ADF Test for the Variables of Raw Data in Group 2. Table S3, AIC Lag for Variables of Raw Data in Group 2. Table S4, ADF Test for the Variables of Low-Pass Filtered Data in Group 1 (Lag = 1). Table S5, ADF Test for the Variables of Low-Pass Filtered Data in Group 2. Table S6, AIC Lag for Variables of Low-Pass Filtered Data in Group 2. Table S7, ADF Test for the Variables of High-Pass Filtered Data in Group 1 (Lag = 1). Table S8, ADF Test for the Variables of High-Pass Filtered Data in Group 2. Table S9, AIC Lag for Variables of High-Pass Filtered Data in Group 2. Figure S1, Low-pass Filtered Temperature and Lower Grindelwald Glacier Extension.
(DOCX)
