# Supplementary material for: Climate Change and Macro-Economic Cycles in Pre-Industrial Europe
Source: PLoS One. 2014 Feb 7;9(2):e88155. doi: 10.1371/journal.pone.0088155 (PMC3917857; doi:10.1371/journal.pone.0088155)
Supplement: File S1 — This file contains Tables S1–S9 and Figure S1. Table S1, ADF Test for the Variables of Raw Data in Group 1 (Lag = 1). Table S2, ADF Test for the Variables of Raw Data in Group 2. Table S3, AIC Lag for Variables of Raw Data in Group 2. Table S4, ADF Test for the Variables of Low-Pass Filtered Data in Group 1 (Lag = 1). Table S5, ADF Test for the Variables of Low-Pass Filtered Data in Group 2. Table S6, AIC Lag for Variables of Low-Pass Filtered Data in Group 2. Table S7, ADF Test for the Variables of High-Pass Filtered Data in Group 1 (Lag = 1). Table S8, ADF Test for the Variables of High-Pass Filtered Data in Group 2. Table S9, AIC Lag for Variables of High-Pass Filtered Data in Group 2. Figure S1, Low-pass Filtered Temperature and Lower Grindelwald Glacier Extension. (DOCX) [file pone.0088155.s001.docx]

**Supporting Information**

Title: **Climate Change and Macro-Economic Cycles in Pre-Industrial Europe**

Authors: **Qing Pei, David. D. Zhang, Harry F. Lee, Guodong Li**

The GCA proposes a two-variable causal model with two stationary time series, *X_t_* and *Y_t_,* with zero means [[1](#_ENREF_1)]:

(S1) $X_{t}=\sum_{j=1}^{m} a_{j}X_{t-j}+\sum_{j=1}^{m} b_{j}Y_{t-j}+\varepsilon_{t}$.

where, *a* and *b* are the coefficients of the time series. *j* is the data of the time series at time point *j*, and *m* is the length of the time series which is set based on lag. ε is the residue and *t* is the time step.

The Augmented Dickey–Fuller (ADF) test approach controls higher-order correlation by adding lagged difference terms of the dependent variable *Y* to the right-hand side of the regression, which can be written as the following equation [[2](#_ENREF_2)]:

(S2) $DY_{t}=\mu+\delta Y_{t-1}+\beta_{1}DY_{t-1}+\beta_{2}DY_{t-2}+...+\beta_{p}DY_{t-p}+\varepsilon_{t}$,

where

(S3) $DY_{t}=Y_{t}-Y_{t-1}$.

The null hypothesis of the series $\{Y_{t}\}$ has a unit root, that is, *H*_0_: δ=0.

where, *β*, *δ* is the coefficient of the time series. *p* is the data of the time series at time point *p*. *D* means the differencing. ε is the residue and *t* is the time step.

The maximum lag in ADF Test can be chosen based on the following statistical formula (Hayashi 2000), which is also adopted by EViews as a default:

(S4) $Lag_{Max}=\mathrm{int}\left[12\times(\frac{T}{100})^{0.25}\right]$.

where *T* is sample size and *int* means integer.

After applying the ADF test on the stationarity status of each data series, the lag length should be selected for the GCA. In the model, the lags of *X_t_* and *Y_t_* are set equally. Given that the *t* or *F* statistic is only a function, it depends on the correlation between the two variables and the set of conditioning variables. If the lag is set the same, then the same conditioning variables can be included [[3](#_ENREF_3)]. Akaike’s information criterion (AIC) is adopted to determine the appropriate lag length [[4](#_ENREF_4)] as the statistical criteria.:

(S5) $AIC(m)=2m-2\ln(L)\begin{matrix} & 1\leq m\leq m_{\max} \end{matrix}$

where *L* is the maximum likelihood achievable by the model, *m* is the number of parameters of the model, and *N* is the number of data points used in the fit.

*m_max_* is the maximum lag, which can be chosen based on statistical Formula A4. We obtain the *m* for the AIC lag in the ADF test and then apply *m* to set the lag in Formula A1 for the GCA.

**Table S1** ADF Test for the Variables of Raw Data in Group 1 (Lag = 1)

|  | No difference | 1st difference | 2nd difference |
| --- | --- | --- | --- |
| Variables | p | p |  |
| Temperature | 0.000*** | 0.000*** | 0.000*** |
| Precipitation | 0.000*** | 0.000*** | 0.000*** |
| Grain yield | 0.011* | 0.000*** | 0.000*** |
| Grain price | 0.000*** | 0.000*** | 0.000*** |
| Population size | 0.346 | 0.163 | 0.000*** |

*Notes*: Those variables with obvious long-term trends such as, grain price and population size were linearly detrended. All data series were filtered by 40-yr Butterworth low-pass filter prior to statistical analysis. Significance (2-tailed): *p < 0.05, **p < 0.01, ***p < 0.001.

**Table S2** ADF Test for the Variables of Raw Data in Group 2

| Variables | No difference | 1st difference | 2nd difference |
| --- | --- | --- | --- |
| Grain price | 0.093^ | 0.000*** | 0.000*** |
| CPI | 0.087^ | 0.000*** | 0.000*** |
| Real wage | 0.001* | 0.000*** | 0.000*** |
| Grain yield | 0.062^ | 0.000*** | 0.000*** |
| Population size | 0.346 | 0.166 | 0.000*** |

*Notes*: Those variables with obvious long-term trends such as grain production, grain price, CPI, real wage, and population size were linearly detrended. All data series were filtered by 40-yr Butterworth low-pass filter prior to statistical analysis. Significance (2-tailed): ^p < 0.1, *p < 0.05, ***p < 0.001

**Table S3** AIC Lag for Variables of Raw Data in Group 2

| Variables | Difference level | AIC Lag |
| --- | --- | --- |
| Grain price | No difference | 11 |
| CPI | No difference | 2 |
| Real wage | No difference | 9 |
| Grain yield | No difference | 4 |
| Population size | 2nd difference | 0 |

**Table S4** ADF Test for the Variables of Low-Pass Filtered Data in Group 1 (Lag = 1)

|  | No difference |
| --- | --- |
| Variables | p |
| Temperature | 0.000*** |
| Precipitation | 0.000*** |
| Grain yield | 0.000*** |
| Grain price | 0.000*** |
| Population size | 0.000*** |

*Notes*: Those variables with obvious long-term trends such as, grain price and population size were linearly detrended. All data series were filtered by 40-yr Butterworth low-pass filter prior to statistical analysis. Significance (2-tailed): *p < 0.05, **p < 0.01, ***p < 0.001.

**Table S5** ADF Test for the Variables of Low-Pass Filtered Data in Group 2

| Variables | No difference | 1st difference | 2nd difference |
| --- | --- | --- | --- |
| Grain price | 0.002** | 0.204 | 0.000*** |
| CPI | 0.004** | 0.119 | 0.000*** |
| Real wage | 0.007** | 0.000*** | 0.000*** |
| Grain yield | 0.150 | 0.064^ | 0.000*** |
| Population size | 0.109 | 0.023* | 0.000*** |

*Notes*: Those variables with obvious long-term trends such as grain production, grain price, CPI, real wage, and population size were linearly detrended. All data series were filtered by 40-yr Butterworth low-pass filter prior to statistical analysis. Significance (2-tailed): ^p < 0.1, *p < 0.05, **p<0.01, ***p < 0.001

**Table S6** AIC Lag for Variables of Low-Pass Filtered Data in Group 2

| Variables | Difference level | AIC Lag |
| --- | --- | --- |
| Grain price | No difference | 15 |
| CPI | No difference | 15 |
| Real wage | No difference | 15 |
| Grain yield | 1st difference | 15 |
| Population size | 1st difference | 15 |

**Table S7** ADF Test for the Variables of High-Pass Filtered Data in Group 1 (Lag = 1)

|  | No difference |
| --- | --- |
| Variables | p |
| Temperature | 0.000*** |
| Precipitation | 0.000*** |
| Grain yield | 0.000*** |
| Grain price | 0.000*** |
| Population size | 0.000*** |

*Notes*: Those variables with obvious long-term trends such as, grain price and population size were linearly detrended. All data series were filtered by 40-yr Butterworth low-pass filter prior to statistical analysis. Significance (2-tailed): *p < 0.05, **p < 0.01, ***p < 0.001.

**Table S8** ADF Test for the Variables of High-Pass Filtered Data in Group 2

| Variables | No difference |
| --- | --- |
| Grain price | 0.000*** |
| CPI | 0.000*** |
| Real wage | 0.000*** |
| Grain yield | 0.000*** |
| Population size | 0.000*** |

*Notes*: Those variables with obvious long-term trends such as grain production, grain price, CPI, real wage, and population size were linearly detrended. All data series were filtered by 40-yr Butterworth low-pass filter prior to statistical analysis. Significance (2-tailed): ^p < 0.1, *p < 0.05, **p<0.01, ***p < 0.001

**Table S9** AIC Lag for Variables of High-Pass Filtered Data in Group 2

| Variables | Difference level | AIC Lag |
| --- | --- | --- |
| Grain price | No difference | 8 |
| CPI | No difference | 3 |
| Real wage | No difference | 10 |
| Grain yield | No difference | 10 |
| Population size | No difference | 10 |

**Figure S1** Low-pass Filtered Temperature and Lower Grindelwald Glacier Extension. The Grey Line is Low-pass Filtered Temperature by 40 year and Black Line is Glacier Extension [[5](#_ENREF_5)]. In the meantime, the result of correlation analysis between low-pass filtered temperature and glacier fluctuations is 0.499, which is significant at the level of 0.1 and justifies that low-frequency change in temperature. Generally speaking, if the temperature is lower, the glacier will advance more, and vice versa as the figure below.


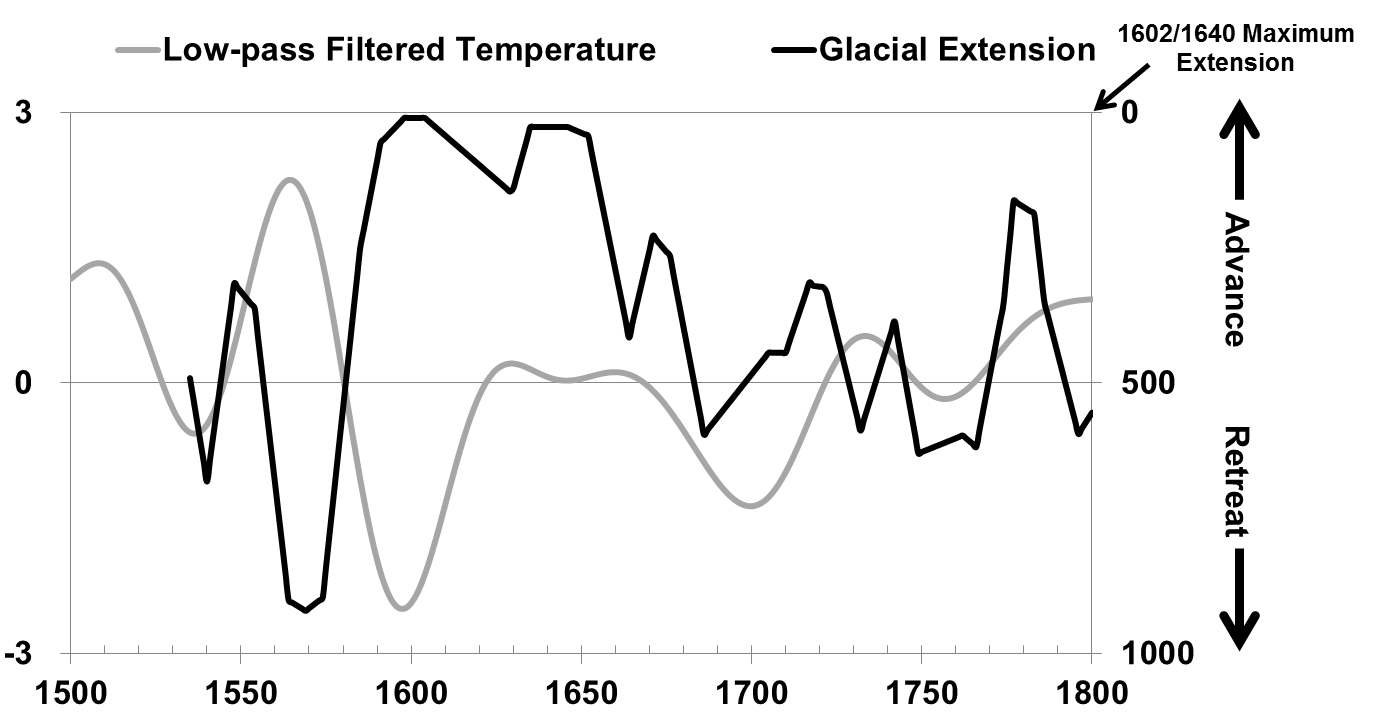


**References of Supporting Information**

1. Granger CWJ (1969) Investigating Causal Relations by Econometric Models and Cross-spectral Methods. Econometrica 37: 424-438.

2. Agung IGN (2009) Time Series Data Analysis Using Eviews. Singapore: John Wiley & Sons (Asia) Pte Ltd.

3. Kirchgässner G, Wolters J (2007) Introduction to Modern Time Series Analysis. Verlag Berlin Heidelberg: Springer.

4. Akaike H (1974) A new look at the statistical model identification. IEEE Transactions on Automatic Control 19: 716-723.

5. Holzhauser H, Magny M, Zumbuhl HJ (2005) Glacier and lake-level variations in west-central Europe over the last 3500 years. The Holocene 15: 789-801.
